# Effect of temperature on evaporation dynamics of sheep's blood droplets and topographic analysis of induced patterns

**DOI:** 10.1016/j.heliyon.2022.e11258

**Published:** 2022-10-28

**Authors:** Ahmad Jaber, Romain Vayron, Souad Harmand

**Affiliations:** University Polytechnique Hauts-de-France, LAMIH, CNRS, UMR 8201, F-59313 Valenciennes, France

**Keywords:** Evaporation of blood drops, Topographic analysis, Marangoni effect, Induced pattern

## Abstract

To characterize various induced phenomena and the blood of healthy sheep using several parameters, the evaporation dynamics of 72 drops of sheep blood evaporated at several temperatures: 23, 37, 60, and 90 °C on glass hydrophilic substrates were studied. This allows the prediction of the sheep blood pattern, knowing the surface temperature and vice versa. To determine the variation in the Marangoni number between the center and the triple line, an infrared thermography method was used to measure the temperature variation along the surface of the drop. Simultaneously, a high-performance camera was used to measure the variation in the height of the drop during the evaporation using a superior algorithm software for image analysis, drop shape analyzer, under controlled conditions (Humidity = 40%, T_atm_ = 23 °C). The study of the evaporation dynamics and pattern formation shows the effect of temperature on the flow circulation inside the drop, resulting in the final deposit. The results showed two categories corresponding to two different evaporation phenomena induced by the thermal Marangoni effect. Furthermore, to transform the induced pattern of sheep blood evaporation into a 3D image, a topographic study was performed using a highly accurate, fast, and flexible optical 3D measurement system. The topographic parameters were subsequently extracted from these 3D images. The statistical study showed a good correlation between the topographic parameters and the surface temperature, and a significant difference between each temperature group for each parameter.

## 1 Introduction

Currently, the detection of diseases and blood characteristics based on the evaporation of blood drops is at the core of research (acoustics [1], fluid dynamics, and thermal analysis [2]). Blood contains myriad information about human health, which can help us to characterize it and provide medical diagnosis. Many studies are underway to understand different morphological and pathological relationships (AMI [1] and pattern geometry [2]). In this study, the effect of surface temperature on dynamic evaporation was investigated, and a new method of blood characterization was developed. A sessile droplet is a liquid droplet that is deposited on a solid substrate where its contact line limits the wet contact area between the liquid and the solid surface and is characterized by the height of the droplet (H), the radius (R), and the contact angle (θ). The droplet evaporates if the atmosphere around its interface is not saturated with its vapor.

Many applications use the evaporation of sessile droplets such as:

• Medical diagnostic techniques: Evaporation of a sessile droplet of cow's milk, blood, and serum. Yakhno et al. tested milk, whole blood, and serum samples from control, bovine leukosis virus positive, and bovine tuberculin-positive cattle groups to provide diagnosis from complex fluids, using biological fluids from healthy and infected cattle, and, using drop drying technology, they found significant differences between them [3]. Evaporation of a droplet of aqueous lysozyme (a globular protein present in saliva) [4]. By evaporating human serum albumin (a globular protein present in blood plasma), Sett et al. developed a rapid methodology to quantify the β-sheet content in human serum albumin [5].
• Cooling microelectronic components: Using dielectric coolants, Estes et al. compared two types of cooling: free jets and spray, and found that spray drops produce much greater critical heat flux at low subcooling than jet cooling [6].
• Nanotechnologies: Dan et al. evaporated a drop of diluted gold nanoparticles suspensions to design materials self-assembled multifunctional optoelectronics [7].

The evaporation of biofluids shows different induced patterns according to the process of self-organization during evaporation, which may be a significant improvement in the diagnosis and detection of diseases. These biofluids include blood serum [1, 8, 9, 10, 11], whole blood [12, 13, 14], and tear fluid [15].

Some studies have shown the effect of temperature on the induced patterns. Parsa et al. reported different types of patterns induced by drying CuO-water nanofluid droplets on substrates heated at different temperatures [16]. This study shows three different types of induced patterns based on three classes of temperature related to the thermal Marangoni effect. At 25 °C, the highest density of nanoparticles was present on the first contact line, and the central region mainly occupied droplet nanoparticles. A double ring phenomenon was observed in the presence of two rings between 47 °C and 81 °C. A stick-slip pattern was observed in the presence of several rings at 99 °C. Patil et al. reported two types of patterns induced by drying a sessile water droplet containing colloidal particles: a ring-like pattern for an unheated substrate and a thinner ring with an inner deposit for the heated substrate caused by the Marangoni effect [17].

The Marangoni effect is a material transport phenomenon that occurs along an interface under the effect of a surface tension gradient. The presence of a surface tension gradient naturally forces the liquid to flow from regions of low tension to regions of high surface tension. This gradient can be caused by the concentration or temperature gradient. Owing to the mixing of two fluids with different surface tensions, solutal Marangoni flow is present [18]. Chen et al. reported the solute Marangoni effect by mixing water with butanol [19], and Parsa et al. reported the thermal Marangoni flow inside binary-based CuO nanofluid droplets evaporated at 64 and 81 °C [16]. Pyeon et al. developed a new coating method to avoid coffee ring pattern formation and found two different Marangoni flows inside the droplets driven by the solutal Marangoni effect according to the droplet geometry [20].

In this study, the effect of temperature during the evaporation of a drop of sheep's blood on the dynamics of evaporation was analyzed by measuring the variation in droplet volume and height as a function of time, and by calculating the number of thermal Marangoni waves between the center and the drop contact line as a function of time. The different induced patterns could be transformed into 3D parameters by studying the topography of their surfaces. This type of analysis is used to easily find the differences between the different induced patterns and characterize each pattern. These parameters are included in the ISO 25178 and EUR 15178N standards [21], specially developed for analysis at the microscale. The surface topography datasets were well characterized by ISO 25178, providing an important reference for the definition of standard procedures and parameters. Thus, during the last few years, the number of studies reporting roughness parameters conforming to this standard has increased at a high rate [22]. Blood was characterized by a topographic study of the induced pattern using roughness and pattern parameters. Finally, we performed a statistical study of these parameters to differentiate the blood patterns.

## 2 Material and methods

Seventy-two drops of sheep blood obtained from the blood Bank of ENVA (école nationale vétérinaire d'Alfort) in collaboration with CRBM (centre de recherche biomédicale), were considered and evaporated at several temperatures: 23, 37, 60, and 90 °C on glass hydrophilic substrates to understand the evaporation dynamics.

### 2.1 Experimental device for studying the dynamics of evaporation

**Figure 1:**
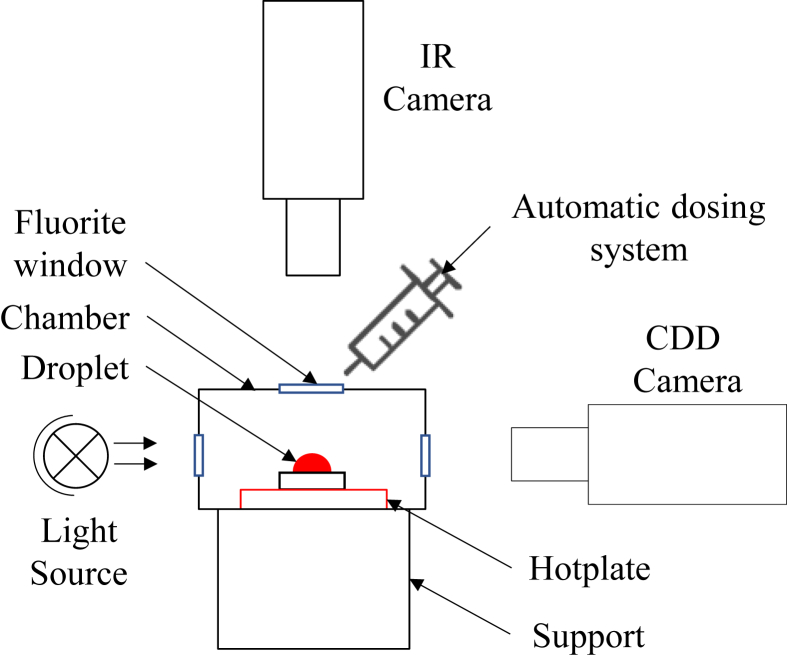
Experimental setup of the Kruss.

The principle of this experiment comprises depositing a drop of sheep blood (heated at T = 21 °C) on a 1 mm thick glass substrate using a software-controlled dosing unit (Kruss, DS4210 Model, Germany), this automatic dosing system produces a reproducible drop volume. The glass was then placed on a heating plate in a controlled goniometer chamber (Kruss, DSA30S Model, Germany). Above the drop, an infrared (IR) camera was installed to measure the temperature variation between the edge and center of the drop to calculate the thermal Marangoni number. In the horizontal plane, a CCD camera (Guppy PRO, 780 × 580 pixels, 62 fps) recorded videos of the evaporation of blood drops using a light source. First, the chamber was set to a constant temperature and humidity (23 °C, 40%). Once the glass substrate reached the desired temperature, a drop was deposited on this surface using the automatic dosing system of the goniometer. The chamber remained closed, and two cameras (CCD and IR) recorded videos and collected data during the evaporation of the drop. The drop shape analyzer software (DSA4) could measure the evolution of the volume, contact angle (right and left), height, and diameter of the base as a function of time, according to this video (see Figure 1).

#### 2.1.1 Marangoni number calculation

The Marangoni effect refers to the transport of matter along an interface under the effect of a surface tension gradient.

Marangoni-type flows can be induced by not only a local change in concentration [20, 21] (solutal Marangoni) but also temperature gradients [22, 23] (thermal Marangoni). Indeed, the surface tension is strongly influenced by the temperature; a temperature gradient along the surface of a drop induces a capillary flow.

The temperature distribution on the surface of a drop and the presence of the Marangoni effect in a drop of sheep blood are discussed. Two types of temperature fields exist on the surface of a drop that evaporates on a heated or unheated (Ts = 23 °C) substrate.

**Figure 2:**
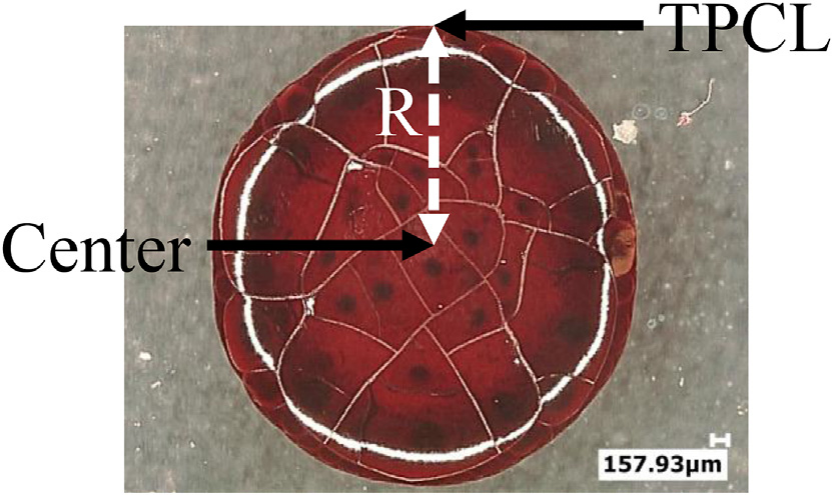
Drop of sheep's blood evaporated at temperature 23 °C. R corresponds to the radius of the drop.

The Marangoni number (Eq. 1) was calculated between the center of the drop and the triple point of the contact line (TPCL) (see Figure 2) as a function of time for temperatures to explain dome formation during evaporation.

Calculation of Marangoni number [27]: $Ma=-\frac{\partial \sigma }{\partial T}\frac{R\Delta T}{\eta \alpha }$

where

(1) $$\alpha =\frac{\lambda }{\rho .C_{p}}$$

Where $\frac{\partial \sigma }{\partial T}$ is the mean of the derivative of the surface tension expression in the TPCL and center. ΔT is the temperature difference on the drop surface between the TPCL and center. α is thermal diffusivity, 0.000137 m^2^/s. The density (ρ = 1.04873 g/ml) was measured using a high-capacity microbalance-type Sartorius by calculating the mean of 10 tests using 3 ml of sheep blood, and the relative uncertainty of the measurement was less than 0.1%. λ is the thermal conductivity, 0.52 w/m/C. Cp is the specific heat capacity, which is equal to 3617 J/kg/°C.

Synder [28] studied the influence of temperature and hematocrit ratio on blood viscosity and found a relationship between the relative blood viscosity, temperature, and hematocrit level (Eq. 3). The relative viscosity of the blood was determined using the following expression (Eq. 2):

(2) $$\eta_{r\;blood}=\frac{\;S_{wp,20\;{}^{\circ}\;C}\;}{S_{Bp}}$$

where $S_{wp}$ is the slope of the water pressure flow lines, and $S_{Bp}$ is the slope of the blood pressure-flow lines.

(3) $$\eta_{r\;blood}=2.03\;\exp\left[\left(0.0332-1.08\times 10^{-4}T\;\right)Ht+0.02T\right]$$

Ht is the hematocrit level of sheep blood, and T is the temperature of the blood.

The viscosity of water [29]:

(4) $$\eta_{water}=2.414\times 10^{\;\frac{247.8}{T+133}-5}$$

To calculate the viscosity of blood, the relative viscosity of blood (Eq. 3) was multiplied by the viscosity of water (Eq. 4).

#### 2.1.2 Surface tension as a function of temperature

**Figure 3:**
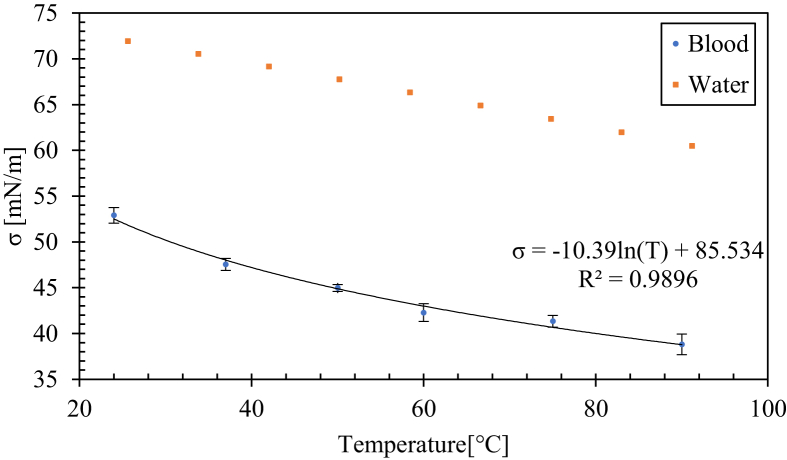
Variation of the surface tension of blood and water as a function of temperature.

The calculation of the Marangoni number requires finding the expression of the surface tension as a function of the temperature for this purpose, the Kruss was used. The chamber was heated to six different temperatures (24, 37, 50, 60, 75, and 90 °C), and then the surface tension was measured for each one. The relationship between the surface tension (σ) and temperature of sheep blood can be determined by plotting the logarithm tendency curve, which has the highest correlation coefficient value (see Figure 3). The relative uncertainty of the measurements was between 0.33 and 1.13% for each temperature.

#### 2.1.3 Measurement of temperature variation

The temperature variation at each point of the drop can be measured as a function of time using an IR camera, the FLIR X6580sc Model (640 × 512 pixels, 15 μm detector pitch) installed above the drop (see Figure 5).

**Figure 4:**
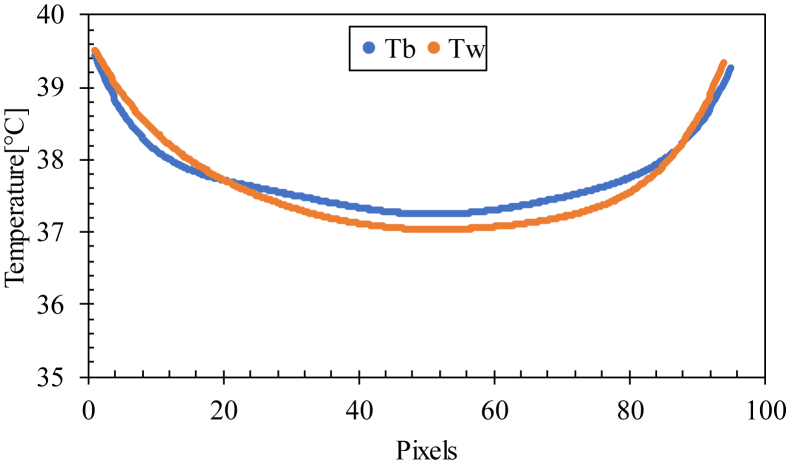
Temperature profile of a line through the center of blood (Tb) and water (Tw) droplets.

The emissivity of blood was close to that of water (ε = 0.95). The temperature profiles of droplets of water and blood evaporated at Ts = 40 °C were compared at the beginning of evaporation, and it was found that the temperature profiles of both were almost the same (Figure 4). A slight difference was observed between the temperature of two fluids at the center (0.2 °C) due to the small differences between the thermal conductivity of these two fluids (0.60 W/m.C for water and 0.52 W/m.C for blood [30]).

**Figure 5:**
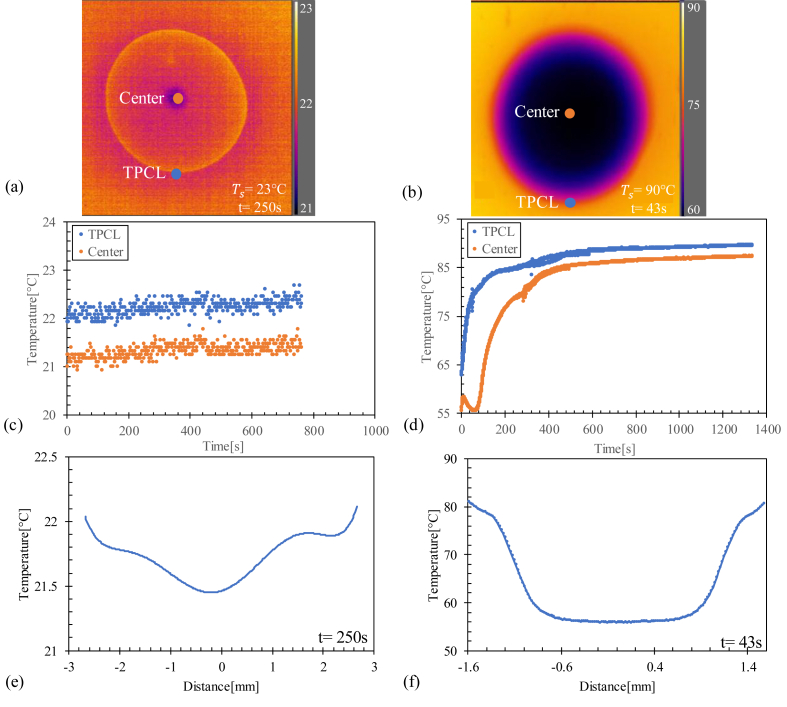
The thermal data extracted by the IR camera from two drops of sheep's blood evaporated at room temperature (left column) (Ts = 23 °C, where Ts is the surface temperature) and Ts = 90 °C (right column). **(a)** and **(b)** are the images of the temperature distribution on the drops at t = 250 s and t = 43 s, respectively. **(c)** and **(d)** represent the variation of the temperature at the TPCL and in the center of the drop as a function of time. **(e)** and **(f)** represent the temperature profile of a line passing through the center of the drop.

### 2.2 Experimental device for topographic analysis

**Figure 6:**
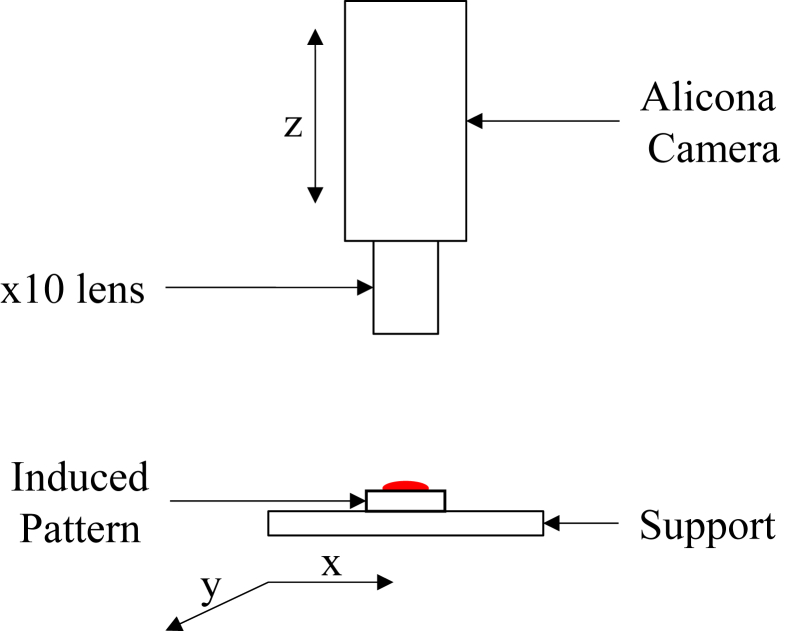
Alicona device.

The principle of these experiments is to topographically map the induced patterns of evaporated blood drops using an Infinite focus G5 device (Alicona, Austria) (see Figure 6), based on the focus variation method. This method combines the shallow depth of an optical system field with vertical scanning to provide topographic information from the change in focus. The data acquired from the device were transformed into 3D information using algorithms, by analyzing the variation in the focus along the vertical axis (z).

A x10 lens was used for these measurements, and the software was forced to automatically divide the drop into elementary sketches (set of images defining the drop, 1.62 mm × 1.62 mm each), and subsequently group them into a single image. The support moved on the XY plane over an area of 100 mm × 100 mm very precisely, so that the camera captured the data of each sketch, with a vertical resolution of 100 nm and a lateral resolution of 1.76 μm.

Topographic parameters were extracted from the 3D images using a topographic analysis software (Mountains®, Digital Surf). These topographic parameters were classified into two groups:36 roughness and 20 pattern parameters. Each parameter was studied as a function of temperature, volume, and diameter.

#### 2.2.1 Roughness parameters

Roughness parameters [23] were divided into 6 subgroups:

• Height parameters: Height parameters are a class of surface texture parameters used to quantify the z-axis perpendicular to the surface. They are included in the ISO 25178 standard published in 2012, which defines areal surface texture parameters.
• Functional parameters: Functional parameters were calculated from the Abbott-Firestone curve, which was obtained by integrating the height distribution over the entire surface.
• Functional volume parameters: Functional volume parameters are typically used in tribological studies. They were calculated using the Abbott-Firestone curve (surface lift rate curve) over the surface.
• Spacing parameters: The spacing parameters describe topographic features based on spectral analysis.
• Hybrid parameters: Hybrid parameters are a class of surface condition parameters that characterize a criterion that depends on both amplitude and pitch, such as slopes and curvatures.
• Element parameters: Element parameters were derived from the segmentation of a surface into patterns (peak or hollow patterns). Segmentation was performed according to the divide-line algorithm.

#### 2.2.2 Patterns parameters

**Figure 7:**
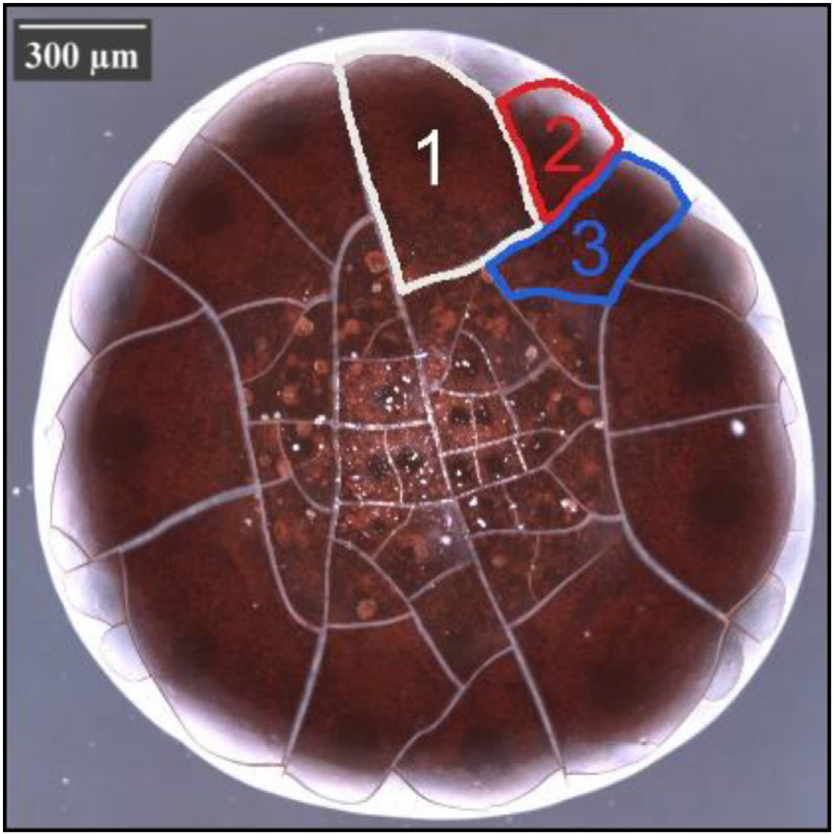
Images of a drop of blood evaporated at room temperature where different patterns can be seen (1, 2, 3, ..., n).

Each sample was composed of several patterns (Figure 7). For pattern parameters, the means parameters of all patterns for each sample were calculated, for example, the “height” parameter is the average height of all the patterns for the same sample ($height=\frac{\sum_{i=1}^{n}{height}_{i}}{n}$).

## 3 Results and discussion

### 3.1 Evaporation dynamics

In this study, the evaporation dynamics of sheep blood drops deposited on a glass substrate at a temperature of 23, 37, 60, 90 °C were analyzed.

#### 3.1.1 Evaporation at Ts = 23 °C

**Figure 8:**
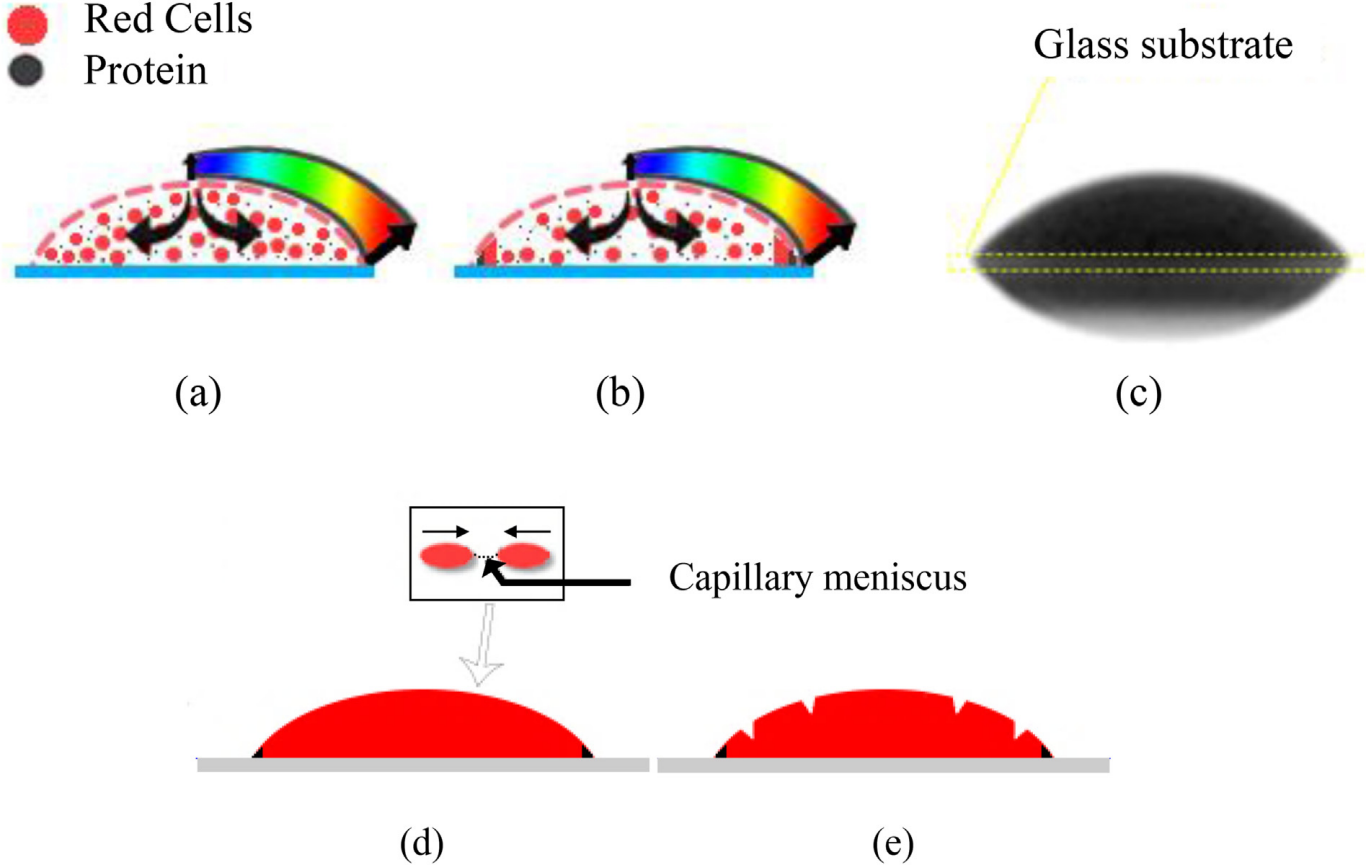
(a,b) Illustration of evaporation of blood drops on a hydrophilic glass substrate. **(c)** Image of a drop of sheep's blood captured by the CCD camera **(d,e)** Schematic of the formation of cracks on a hydrophilic glass substrate.

The drop deposited on the glass substrate at 23 °C spread and wet the surface, as shown in Figure 8. The protein macromolecules of blood adhered to the surface of the glass after the deposition of the drop on the surface [31], which prevented the contact line movement during evaporation (CCR mode).

**Figure 9:**
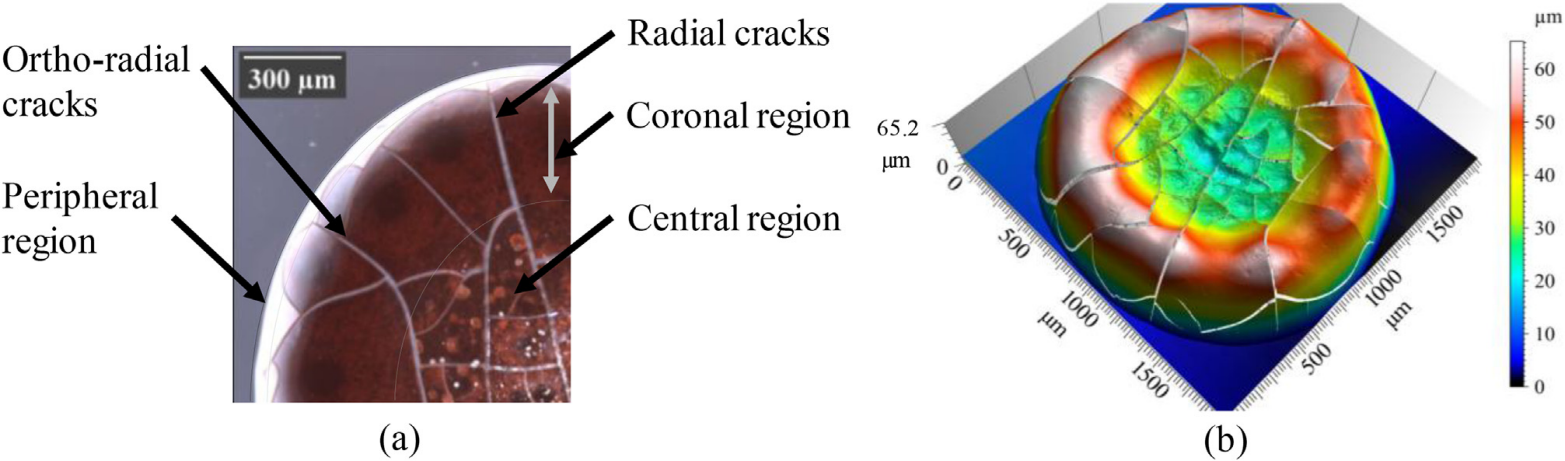
(a) Image of morphological deposit patterns of blood drops on hydrophilic glass substrate. **(b)** 3D morphological deposit image.

The maximum amount of plasma evaporated after a certain time (20 min for a droplet of volume 11 μl for example), then the capillary meniscus (connections between red blood cells, see Figure 8d) was formed among the adjacent red blood cells because the evaporation of the plasma still occurred in the droplet surrounded by cellular components. Subsequently, the red blood cells moved closer to each other by decreasing the radius of curvature of the capillary meniscus. This movement created a compressive stress inside the drop owing to the high capillary pressure generated. The drop released the stresses by forming cracks, where the compressive stress exceeded the resistance of the drop surface, as shown in Figures 8e and 9.

The induced pattern includes three regions as shown in Figure 9a:

• Peripheral region: located at the edge of the drop and without cracks between the grey and black circles.
• Central region: located at the center of the drop with small cracks, between the drop center and the small gray circle.
• Coronal region: located between two regions with radial and ortho-radial cracks.

**Figure 10:**
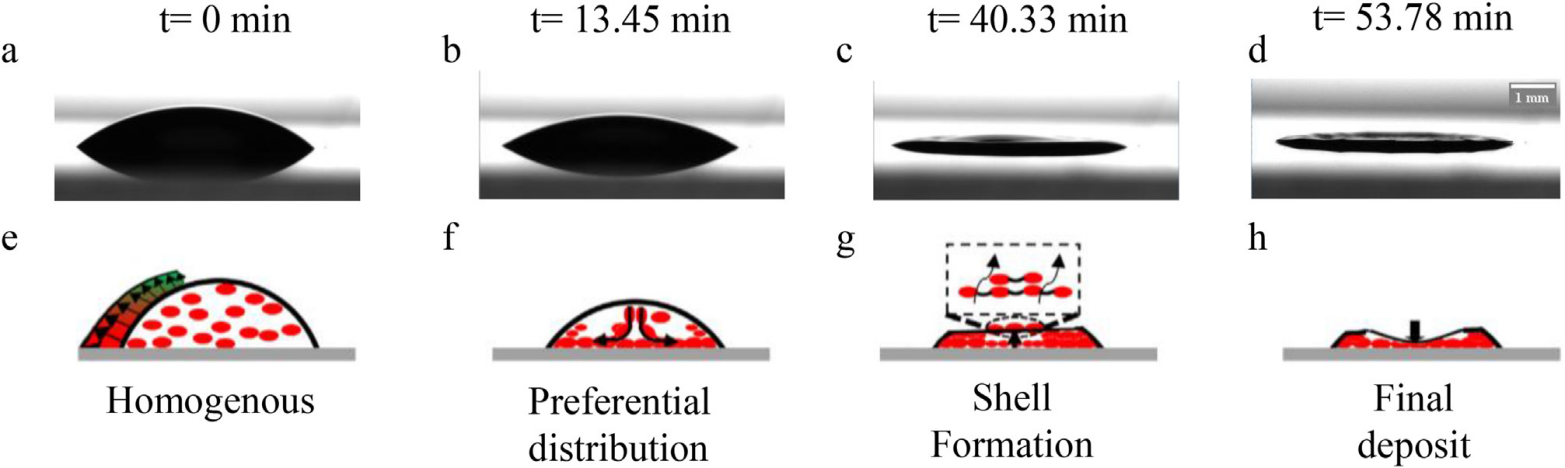
Sequential temporal images of the evaporation of the blood drop on a glass substrate at temperature 23 °C **(a-d)** Side view **(e-h)** Schematic diagram of the dynamics of evaporation inside the drop.

The initial contact angle of the drop of blood was 50 °at t = 0 s. Subsequently, it decreased with the drop height and volume owing to plasma evaporation, as shown in Figure 10(a,b) in the side view. At t = 0 s, the red blood cells (gray ellipses in Figure 10) were homogeneously suspended inside the drop (see Figure 10e). Thus, the non-uniform evaporation flow due to the non-uniform evaporation flux, which was greatest near the TPCL (see Figure 10e), induced an internal flow inside the drop (see Figure 10f), and the red blood cells were deposited on the substrate. Subsequently, the red blood cells moved closer to each other, which increased the concentration of red blood cells simultaneously with the evaporation of the plasma.

After a while, the shape of the drop became almost flat after it was oval (Figure 9c). In addition, the concentration of red blood cells increased at the edge and decreased at the center of the drop, which implies the presence of a cavity in the center and the formation of the coronal region, as shown in Figures 9b and 10h, showing a side view of the final deposit.

**Figure 11:**
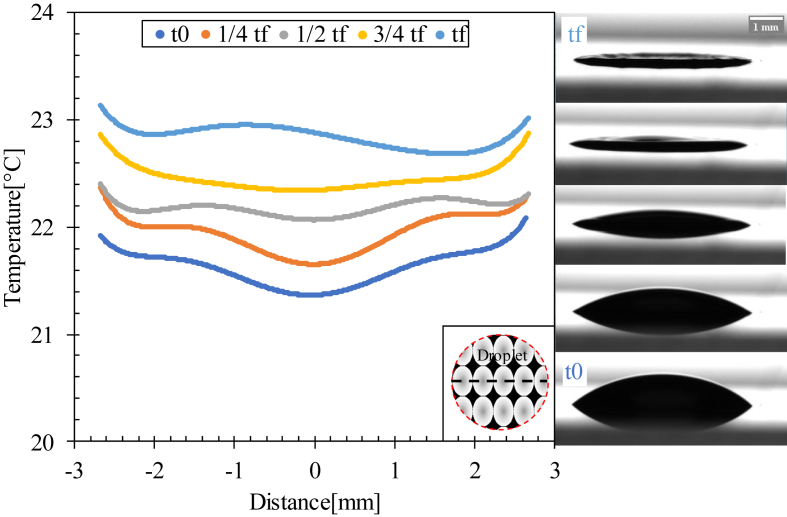
Temperature profiles of a line passing through the center of the drop evaporated at Ts = 23 °C for various time scales. t0 is the time at which evaporation starts and tf is the full evaporation time.

The temperature gradient inside the droplet was extremely low. The nonlinear distribution of temperature induced a buoyant flow inside the droplet between the beginning of evaporation (t0) and the half full evaporation time (1/2 tf), where a non-uniform temperature distribution was detected between the edge and the center of the droplet caused by the solutal Marangoni effect, which created a Marangoni circulation inside the droplet (Figure 11).

The coronal region formation started from half-time evaporation, as shown in Figure 11, due to the capillary flow dominant inside the droplet, which moved the red blood cells and proteins from the center to the edge of the droplet. This flow resulted in a gap in the center and a coronal region at the edge owing to the particle movement from the center to the edge of the droplet.

#### 3.1.2 Evaporation at Ts = 37 °C

**Figure 12:**
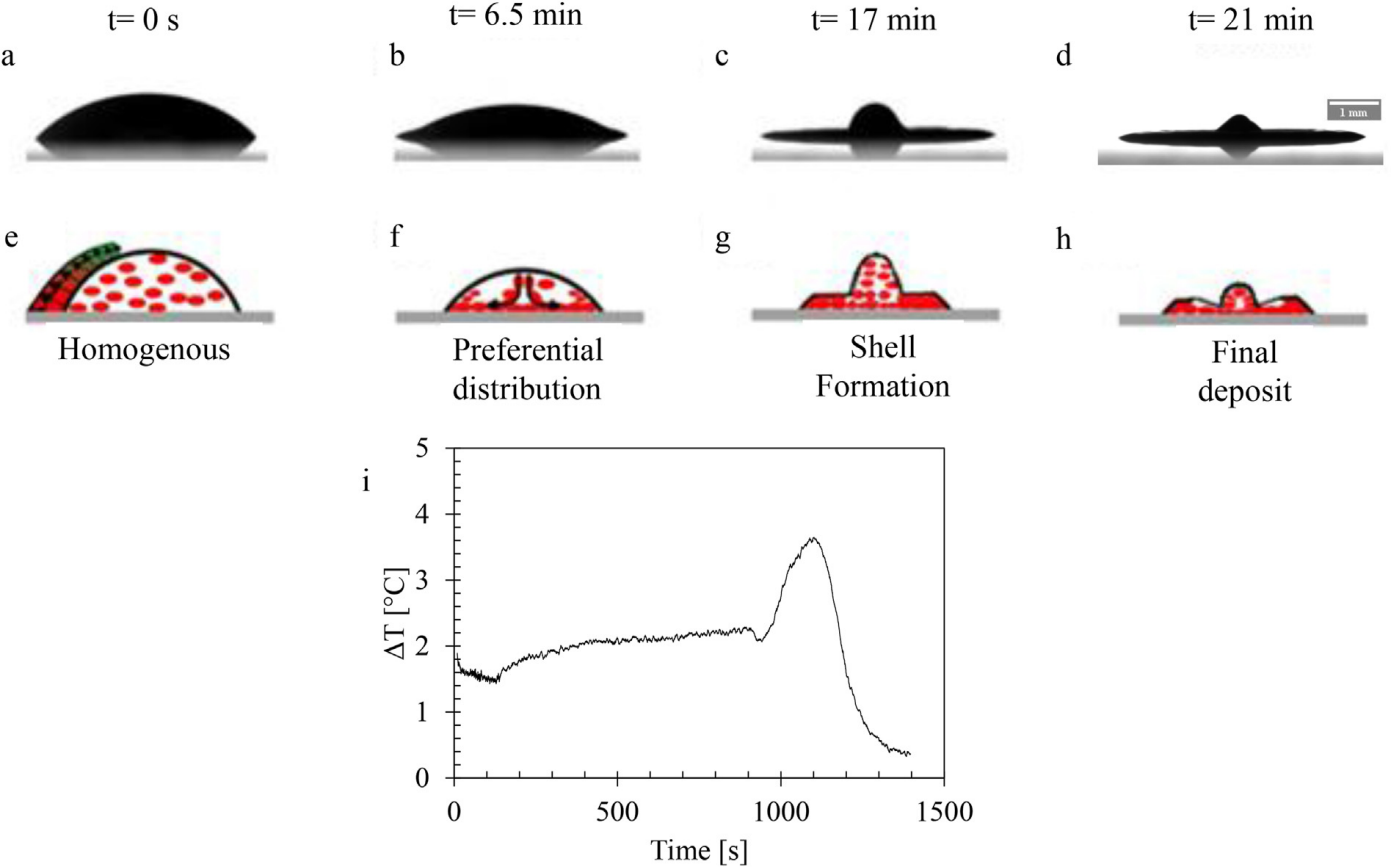
Sequential temporal images of the evaporation of the blood drop on a glass substrate at 37 °C **(a-d)** Side view **(e-h)** Schematic illustration of the dynamics of evaporation inside the drop. **(i)** Variation of the temperature gradient as a function of time.

At the beginning, identical behavior was demonstrated by the drop of blood evaporated at 37 °C and at 23 °C; at 37 °C evaporation, the height of the drop decreased, and the contact radius remained constant. After some time, the temperature gradient between the center and TPCL ($\Delta T=T_{TPCL}-T_{center}$) increased, and a dome formed in the center (see Figure 12).

#### 3.1.3 Evaporation at Ts = 60 and 90 °C

**Figure 13:**
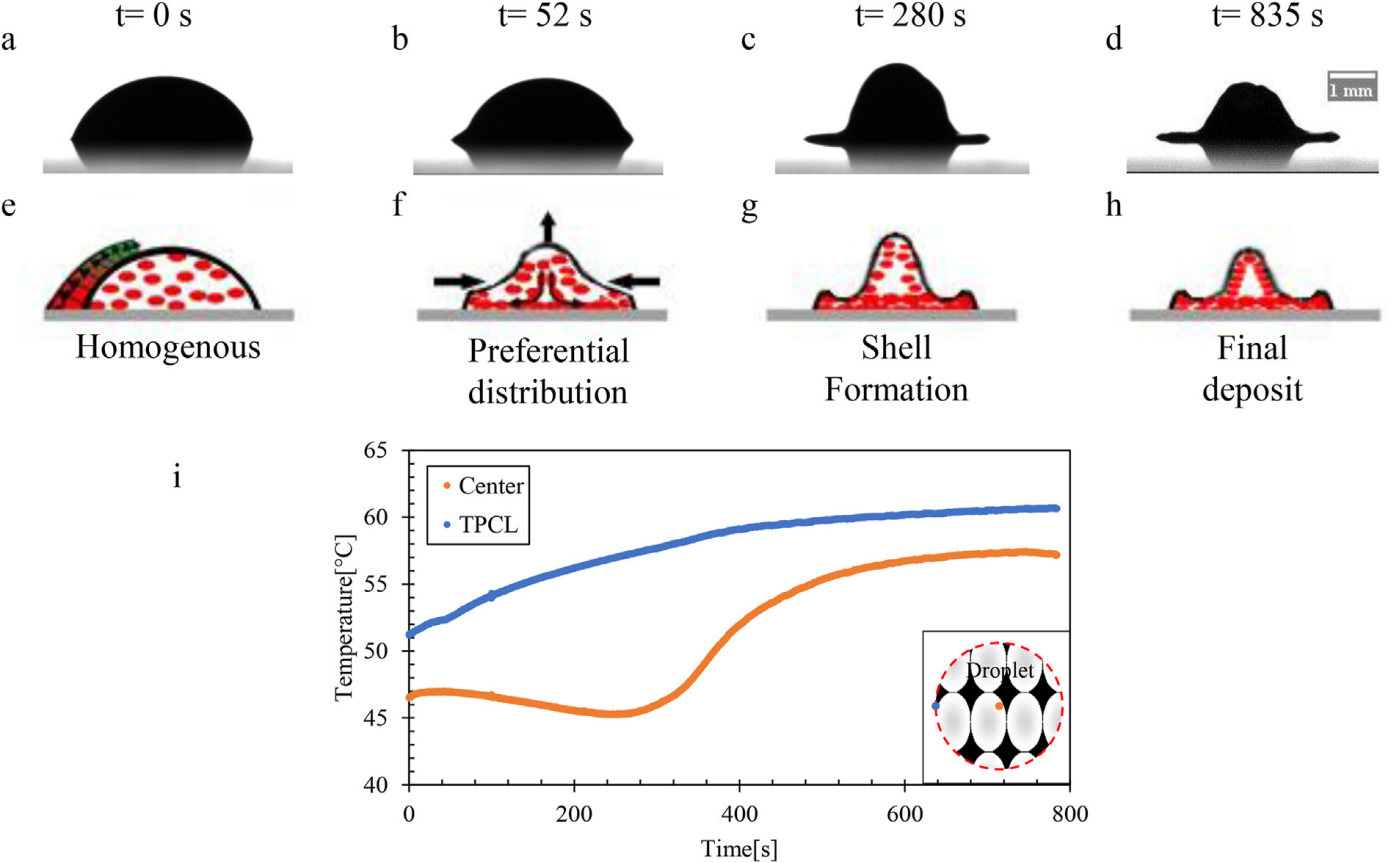
Time sequential images of the evaporation of the blood drop on a glass substrate at temperature 60 °C **(a-d)** Side view **(e-h)** Schematic illustration of the evaporation dynamics inside the drop. **(i)** Temperature variation at TPCL and in the center of the drop as a function of time.

In this section, the effect of temperature on the final deposition of a drop of evaporated blood on a glass substrate is discussed. A significant difference between the induced patterns of a drop of blood evaporated at 23 °C and that evaporated at 60 °C or 90 °C (see Figure 13) was observed.

Partial wetting was observed, and red blood cells and proteins were distributed homogeneously at t = 0 s. The evaporation rate on the TPCL was much greater than that at the center of the drop because of the temperature difference between the TPCL and shells. The volume of this shell decreased as a function of time owing to the evaporation of the plasma remaining inside the drop. Subsequently, the volume decrease ceased, and the formation of cracks commenced.

During the study of the evaporation dynamics of a drop of blood, a change in the evaporation phenomenon was observed with an increase in the temperature and the formation of a dome in the center of the drop. To fully understand this phenomenon, several tests on the evaporation of drops of blood deposited on a hydrophilic substrate (glass), at 23, 37, 60, and 90 °C were performed. The surface tension of the blood drop was measured as a function of temperature to calculate the Marangoni number, and it was found that the drop height increased to form the dome owing to the increase in the thermal Marangoni number (see Figure 15).

#### 3.1.4 Classification of tests

**Figure 14:**
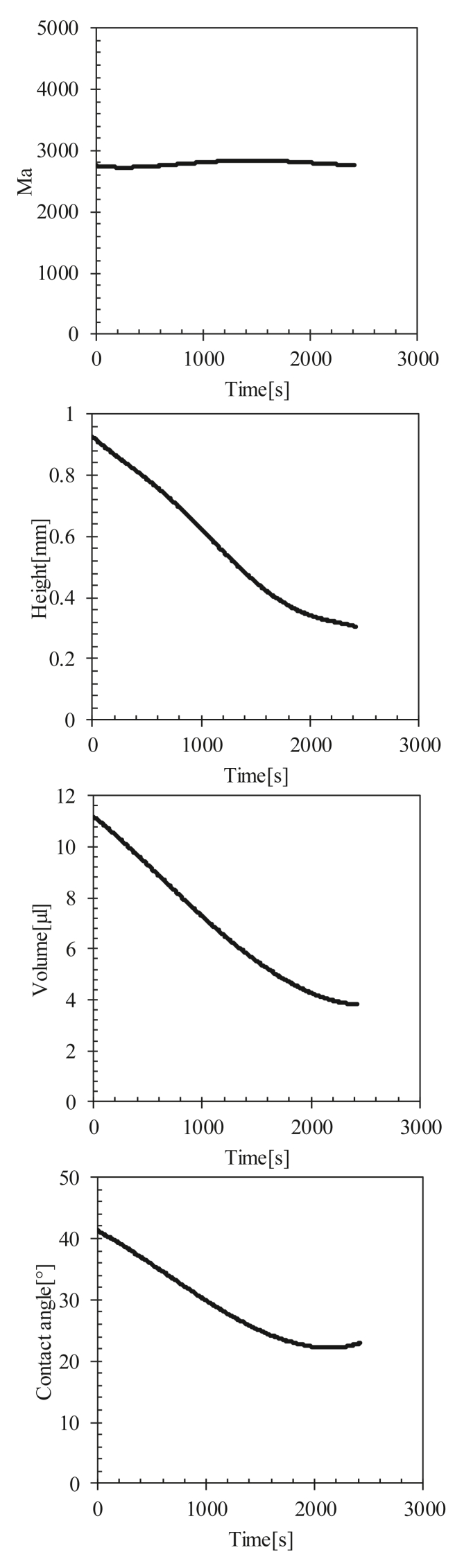
Change in height, volume, contact angle, and Marangoni number of a drop of blood as a function of time (Ts = 23 °C).

Based on our observations, our tests can be divided into two categories:

1 Evaporated test without heating (Ts = 23 °C): All the parameters (volume, height, contact angle) decreased linearly, but the thermal Marangoni number did not undergo significant variation (see Figure 14).

**Figure 15:**
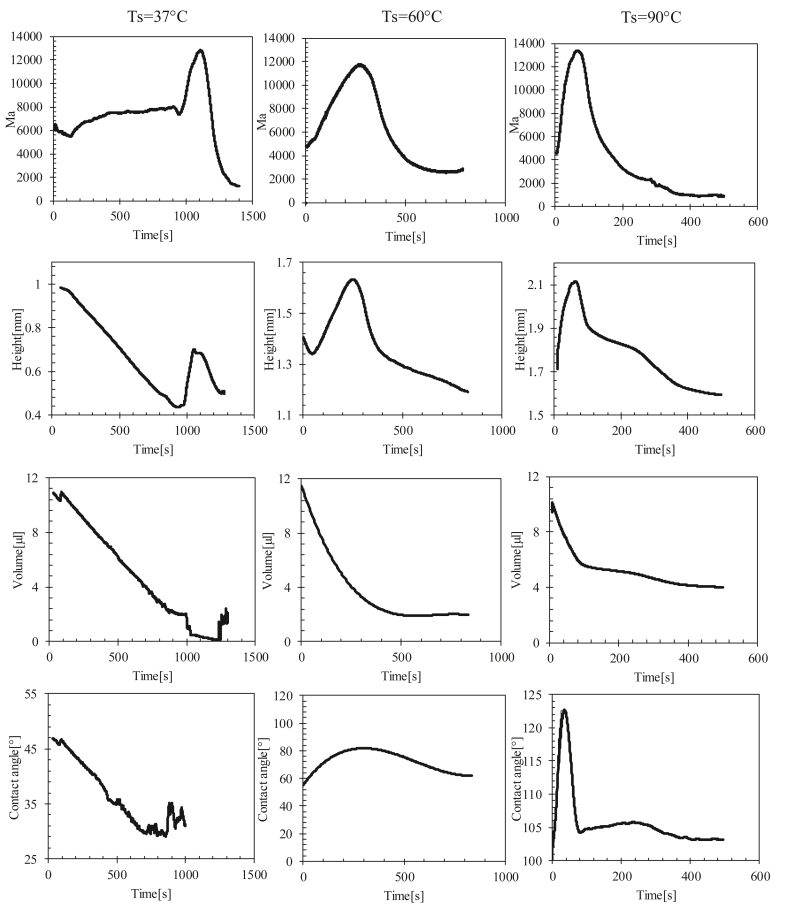
The evolution of the height, volume, contact angle, and Marangoni number for three drops of blood as a function of time.
2 Evaporated test with heating (Ts = 37, 60, 90 °C): The height and the contact angle increased with the increase in the Marangoni number and then decreased with it for Ts = 60 and 90 °C (see Figure 15). For the Ts = 37 °C, a small increase in Ma was observed in the first 900 s of evaporation at a rate of $\frac{{Ma}_{t=900s}-{Ma}_{t=0s}}{900}\approx 1.66$ unit/s. Ma drastically increased at a higher rate ($\frac{{Ma}_{t=1000s}-{Ma}_{t=900s}}{100}\approx 55$ unit/s) after 900 s of evaporation, which was more than 33 times. This increase led to an increase in the height and contact angle of the drop, and therefore, led to the formation of a dome in the middle of the drop.

As shown in a previous study, different evaporation dynamics have been reported under various Ts values. The main reason for this is the change in the flow circulation inside the droplet during evaporation.

For Ts = 23 °C, the capillary flow was dominant inside the droplet, where the Ma slowly increased linearly. In this case, the height, volume, and contact angle of the droplet decreased linearly during evaporation (Figure 14).

**Figure 16:**
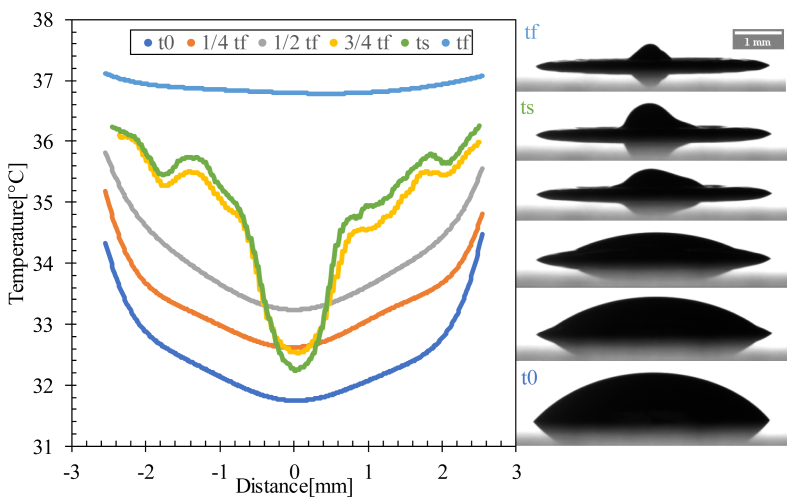
Temperature profiles of a line passing through the center of the drop evaporated at Ts = 37 °C for various time scales. t0 is the evaporation start time, ts is the specific time at which Ma reaches its maximum value, and tf is the full evaporation time.

For Ts = 37 °C, the Ma increased slowly for 15 min, which dominated the capillary flow inside the drop, as well as the evaporated drop at Ts = 23 °C. Ma drastically increased, indicating the presence of a Marangoni circulation inside the drop caused by the temperature gradient created between the center and edge of the droplet (Figures 16 and 12i). This circulation led to the formation of domes by increasing the height of the droplets and contact angle (shown in Figure 15).

**Figure 17:**
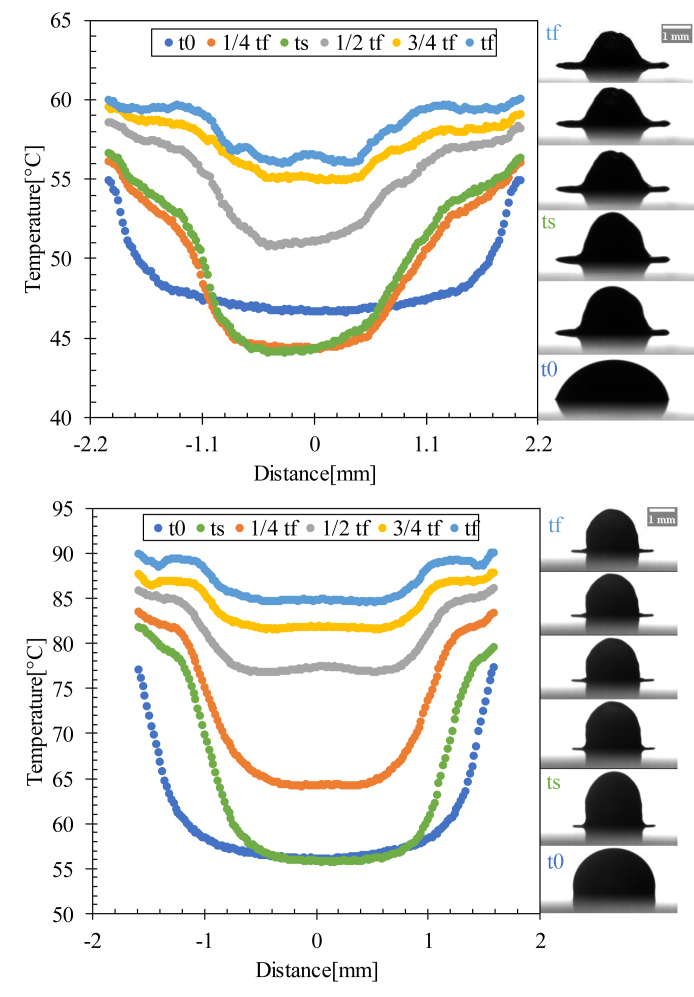
Temperature profiles of a line passing through the center of the drop evaporated at Ts = 60 and 90 °C for various time scales. t0 is the evaporation start time, ts is the specific time at which Ma reaches its maximum value, and tf is the full evaporation time.

For Ts = 60 °C and 90 °C, the Marangoni circulation was dominant, in which a high rate of increase of Ma was observed from the start of evaporation due to the temperature gradient between the center and the edge of the droplet, leading to the formation of domes with an increase in height and the contact angle from the start of evaporation (Figures 15 and 17).

#### 3.1.5 Slope of the volume shrinkage curve

From the aforementioned results, it can be observed that the volume of the drop decreased linearly at the beginning. For this, the slopes of these curves were determined and studied first as a function of the temperature and then as a function of the temperature and volume of the drop (see Appendix Figure 21 and Figure 22). Fifty-eight tests were performed in this study, for which all data were available. The slope increased with the surface temperature. This slope is related to the evaporation rate; the higher the slope, the higher is the evaporation rate.

The values of the regression coefficient were acceptable; therefore, this parameter was used to characterize sheep blood in the remainder of our study.

### 3.2 Topographic study

To characterize sheep blood, variations in 56 patterns and roughness parameters (Table 1) were studied as functions of temperature, volume, and diameter.

**Table 1:** Topographic parameters definitions.

Sq	Standard deviation of the height distribution, or root mean square roughness of the surface
Sp	Maximum peak height. It is the height between the highest peak and the middle plane
Sv	Maximum height of hollows. It is the depth between the middle plane and the deepest valley
Sz	Maximum height. It is the height between the highest peak and the deepest valley
Sa	Mean arithmetic height. It is the roughness of the mean surface, it gives the arithmetic mean, in absolute value, of the difference in height of each point with respect to the mean plane of the surface
Smc	The inverse surface material ratio, is the height [c], which gives the surface material ratio p%
S10z	Ten-point height of surface
S5v	Five-point pit height
Sk	Core roughness. This parameter is calculated as the difference of the heights at the area material ratio values of 0% and 100% on the equivalent line
Svk	Reduced valley depth. This parameter gives the arithmetic mean of the valley depth removed from the surface material ratio curve
Vv	Void volume. Volume of voids at a lift rate p (in %)
Vmc	Material volume of the core of the surface. Volume of material in the core, between two lift rates p and q (in %)
Vvc	The difference between the void volume at the p% value of the surface area ratio and the void volume at the surface area ratio q%
Coplanarity	Maximum vertical distance between the peak of this particle and the peaks of neighboring particles
Circularity	Ratio between the area of the particle and the area of the disc having as diameter the maximum diameter of the particle
Compactness	Ratio between the equivalent diameter and the maximum diameter

First, by studying each temperature separately with good correlations, the relationship between the volume (V) and diameter (D) of the drops was determined, as shown in Figure 14. This relation will be used later to determine the volume of some droplets from the diameter and surface temperature for plotting the variation in topographic parameters versus volume and temperature.

**Figure 18:**
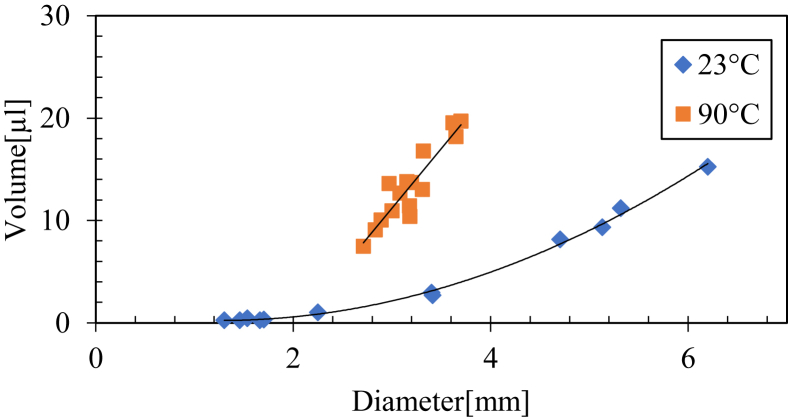
The variation of drop volume as a function of the diameter for the droplet evaporated at 23 °C and 90 °C surface temperature.

Temperature is a critical parameter in the present study and is responsible for the change in the evaporation dynamics of the drop, and therefore for the change in the geometry, as already shown in the previous section. Therefore, the curves of the parameter variations were plotted as a function of temperature (see Appendix Figures 22, 23, and 24), and the linear regression coefficients were calculated for each parameter (see Figure 18).

Sixteen roughness parameters were found to be well-correlated as a function of temperature (see Figure 18). Blood can be characterized using these parameters, and the evaporation temperature can be determined from the roughness parameters.

All parameters were rechecked by plotting 3D curves of the parameters as a function of temperature and volume, as the volume parameter is an important parameter in our study, which affects the final size of the induced pattern. Subsequently, the first order polynomial regression plans were plotted for each parameter to calculate the regression coefficients: degree 11 (degree x = 1 and y = 1) (see Appendix Figures 25, 26, and 27).

**Figure 19:**
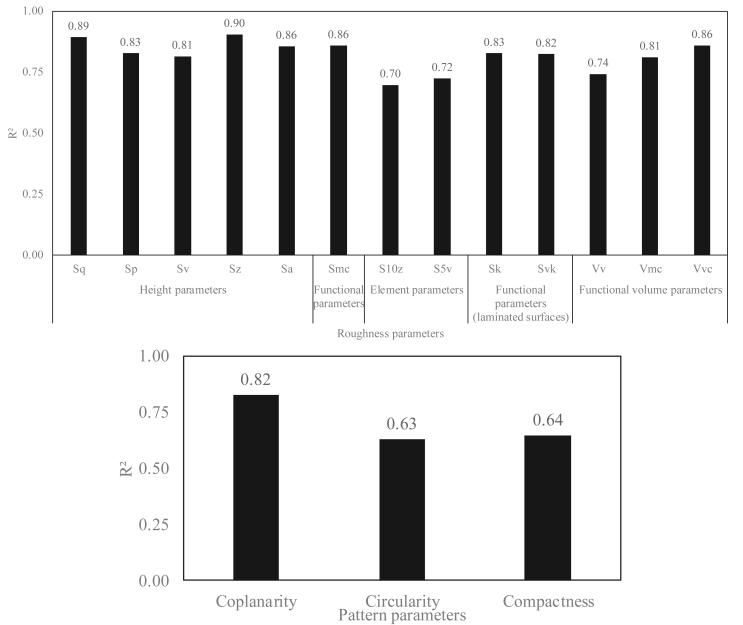
Regression coefficients of the curves of variations of roughness and pattern parameters as a function of the surface temperature.

**Figure 20:**
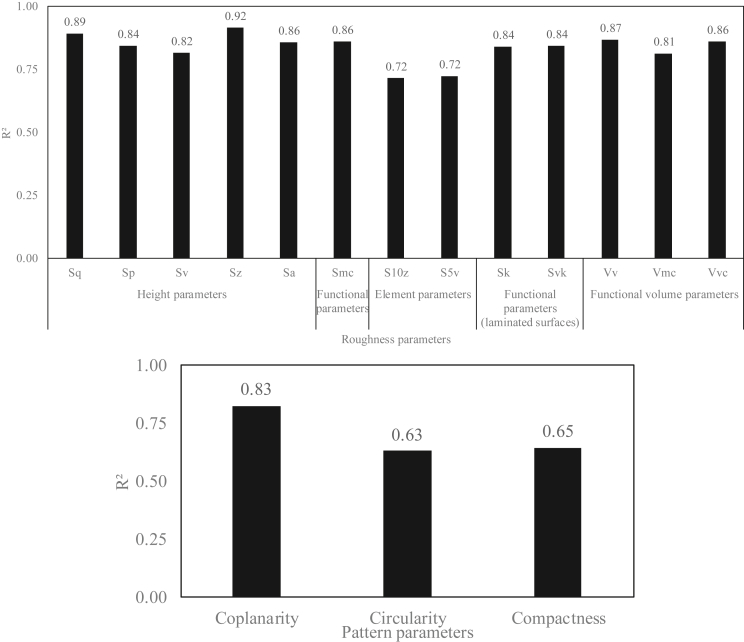
Regression coefficients of the curves of variations of roughness and pattern parameters as a function of the surface temperature and the drop volume.

**Figure 22:**
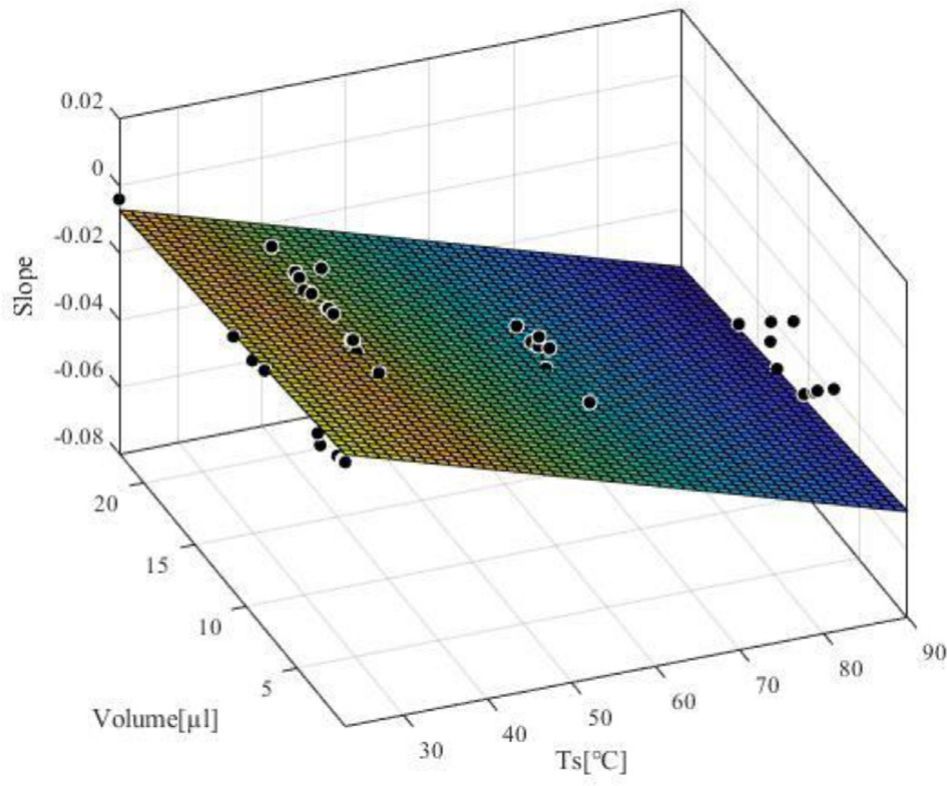
The polynomial regression plane 11 (degree of x = y = 1, R^2^ = 0.89) of the variation in the slope of the volume shrinkage curve as a function of V and Ts.

Better values were found for the regression coefficients by plotting the variation in these parameters as a function of volume and temperature (see Figures 19 and 20).

### 3.3 Statistic study

In this study, data from sheep blood samples (72 tests, 18 tests for each temperature) were used. One-way analysis of variance (ANOVA) was performed. The Tukey-Kramer method was used to compare the values of the parameters at different temperatures (groups). This function provides pairwise comparison results of a multiple comparison test using information from the groups. To determine the error of the ANOVA and Tukey-Kramer statistical tests, a few studies were investigated by changing the distribution index, number of treatments, and sample size via simulation. The exponential distribution using a small number of treatments yielded the largest differences with a deviation of approximately 0.3%, and this value decreased by increasing the number of treatments and the index for the gamma distribution [32]. This method has been used in various applications, and Parikh et al. found significant differences between three types of reline materials with three different types of surface treatments [33]. Hanif et al. found significant differences between demographics, clinical diagnoses, and institutional vascular severity scores [34].

Tukey-Kramer also demonstrated an interactive graph of estimates and comparison intervals. Each group mean was represented by a symbol, and the interval was represented by a line starting from the symbol. The means of the two groups significantly differed if their intervals are disjoint; however, they did not differ significantly when their intervals overlapped.

From the p-value, it was possible to determine whether there was a significant difference between the groups. The results demonstrated a P-value less than 0.05 for all the topographic parameters used in the previous study (see Table 1), thus showing a significant difference between them. The following table (see Table 2) shows the significant differences between each temperature using the arrows; for example, for Sp, there are significant differences across all the temperatures (see Table 3). These results demonstrate the possibility of predicting the evaporation temperature of a drop from the parameters of the induced patterns of the evaporated drop. For some parameters, the mean values for each temperature were very close; no significant difference was found. For example, the parameter Sda has a p-value equal to 0.678, which is greater than the significance level (see Table 4).

**Table 2:** P-values of the topographic parameters, Anova table.

Parameters	P-value
Sq	2.41E-37
Sp	1.64E-31
Sv	7.99E-28
Sz	2.92E-36
Sa	2.03E-35
Smc	2.32E-32
S10z	2.08E-19
S5v	1.39E-21
Sk	8.00E-32
Svk	3.04E-27
Vv	5.57E-33
Vmc	1.95E-32
Vvc	3.87E-32
Coplanarity	9.65E-26
Circularity	2.41E-15
Compactness	6.89E-16

**Table 3:** The significant difference between different surface temperatures for topographic parameters.

Parameters	Ts	Significant difference
Sp, Sv, Sz, S10z, Svk, Copmanirity	23	
	37	
	60	
	90	
Sq, Sa, Smc, S5v, Sk, Vv, Vmc, Vvc	23	
	37	
	60	
	90	
Circularity, Compactness	23	
	37	
	60	
	90

**Table 4:** Sda parameter, ANOVA and Tukey-kramer test.

Parameters	Ts	Mean	STD	Significant difference	P-value
Sda	23	27526.41	13555.45	no significant difference was found	0.68
	37	34602.57	16747.99		
	60	29999.97	17414.79		
	90	31592.98	21830.85		

## 4 Conclusion

We performed a study on the evaporation dynamics of a sheep blood droplet deposited on a heated glass substrate. Experiments were performed in controlled room temperature air using a goniometer device and superior algorithms for image analysis software. As the droplet evaporation tests were repeated for each surface temperature, the same evaporation phenomenon and induced pattern were found. The induced pattern was topographically mapped using a focus-variation device. The roughness and pattern parameters were measured using topographic analysis software. The main conclusion of this work can be summarized as follows:

• The effect of surface temperature on the dynamics of blood evaporation and various phenomena due to the presence of Marangoni flow for drops evaporated on a heated substrate were demonstrated.
• The evolution of the droplet evaporation rate as a function of the surface temperature and the droplet volume was determined by measuring the volume shrinkage slope as a function of time, and this parameter was used to characterize blood.
• Topographic study of all the induced patterns was performed by extracting the roughness and pattern parameters and then selecting 16 parameters that correlated well with the surface temperature and droplet volume to characterize the selected blood.
• Significant differences were found between the temperature classes for all parameters, indicating the possibility of predicting the evaporation temperature of a drop from the parameters of the induced patterns of evaporated drop.

## Declarations

### Author contribution statement

Ahmad Jaber: Conceived and designed the experiments; Performed the experiments; Analyzed and interpreted the data; Contributed reagents, materials, analysis tools or data; Wrote the paper.

Romain Vayron, Souad Harmand: Conceived and designed the experiments; Analyzed and interpreted the data; Contributed reagents, materials, analysis tools or data.

### Funding statement

This research did not receive any specific grant from funding agencies in the public, commercial, or not-for-profit sectors.

### Data availability statement

Data included in article/supp. material/referenced in article.

### Declaration of interest's statement

The authors declare no conflict of interest.

### Additional information

No additional information is available for this paper.

## Figures and Tables

**Figure 21:**
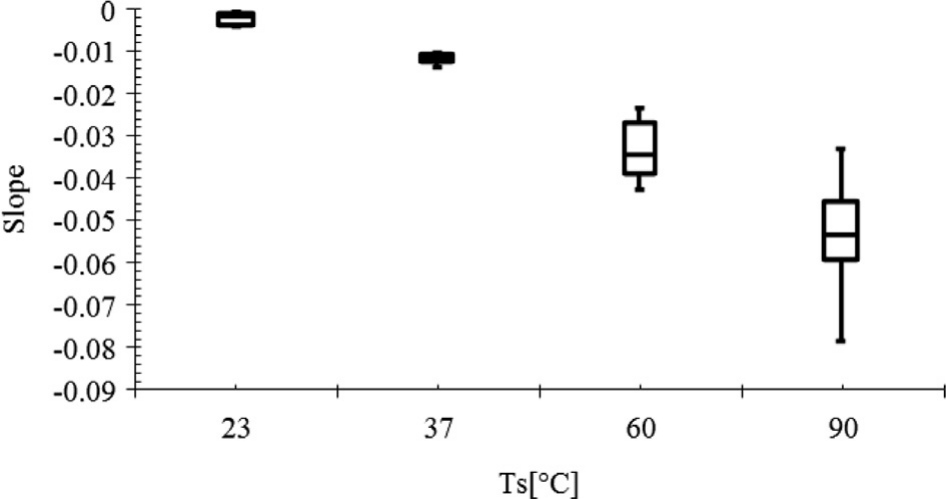
Variation of the slope of the volume shrinkage curve as a function of Ts.

**Figure 23:**
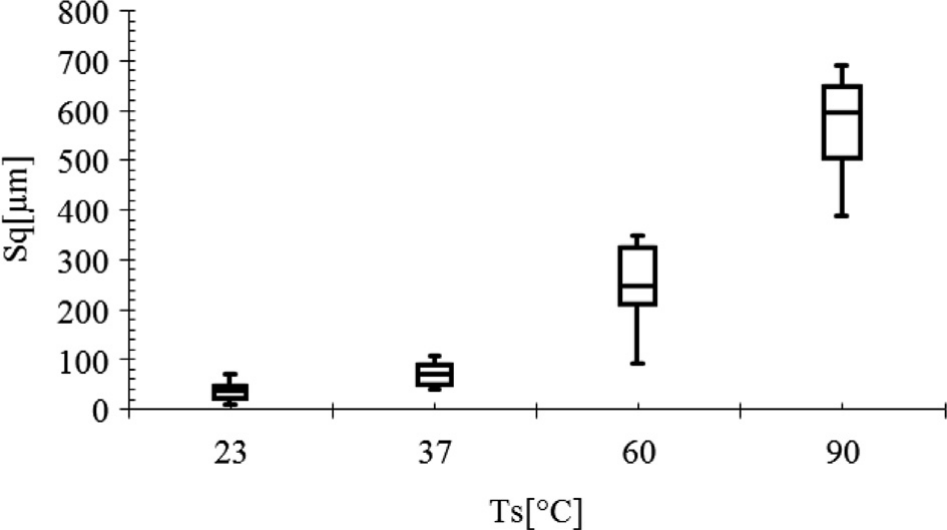
Variation of the Sq parameter as a function of Ts.

**Figure 24:**
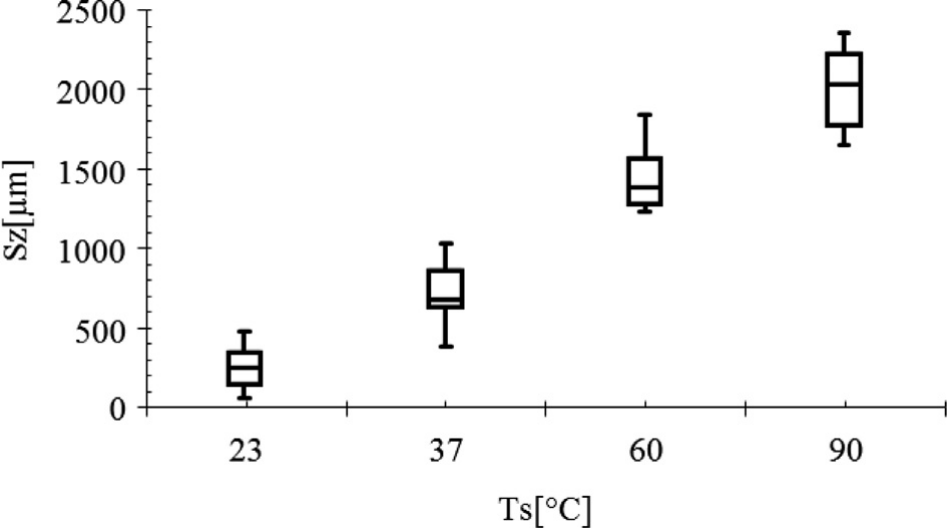
Variation of the Sz parameter as a function of Ts.

**Figure 25:**
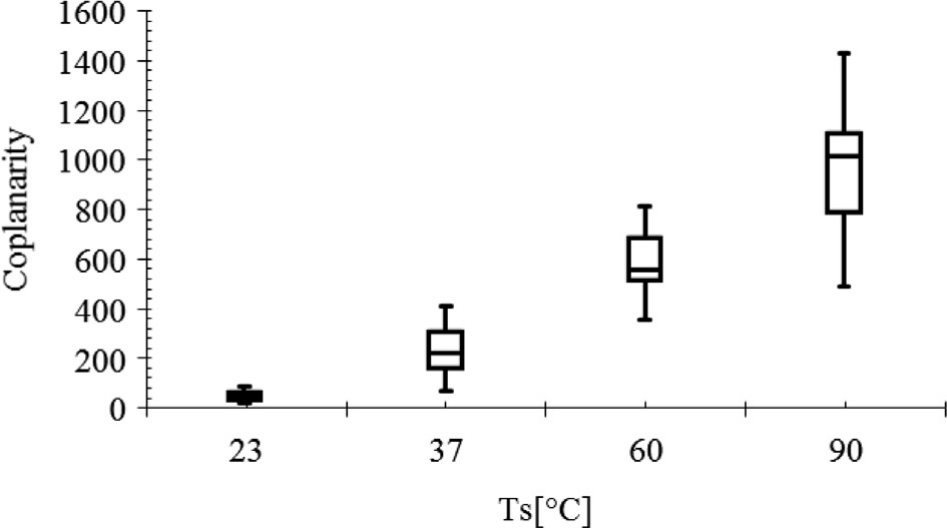
Variation of the Coplanarity parameter as a function of Ts.

**Figure 26:**
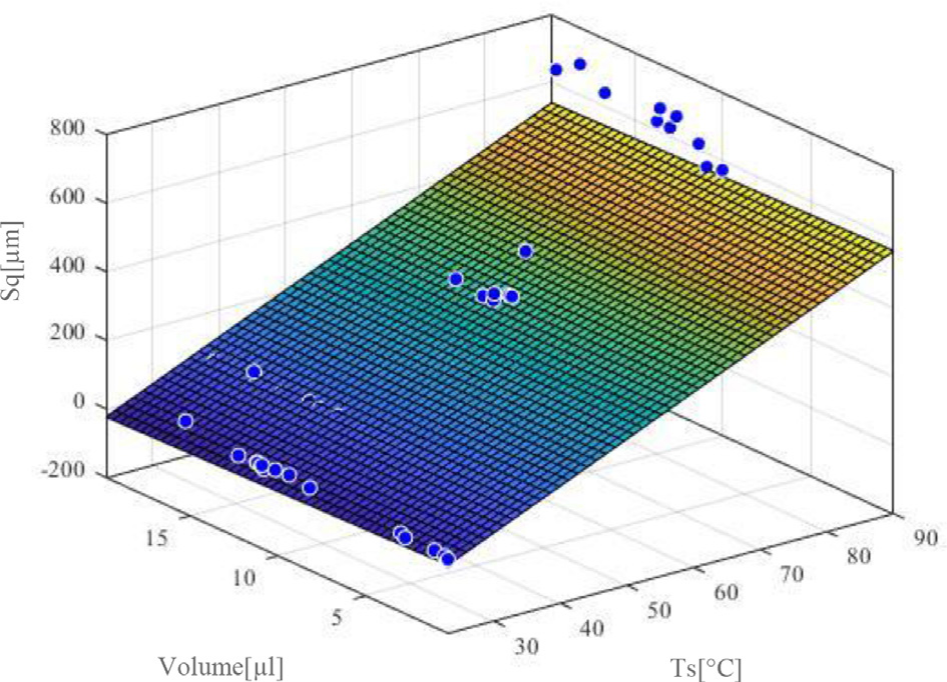
The polynomial regression plane 11 (R^2^ = 0.89) of the variation of the Sq parameter as a function of the V and Ts.

**Figure 27:**
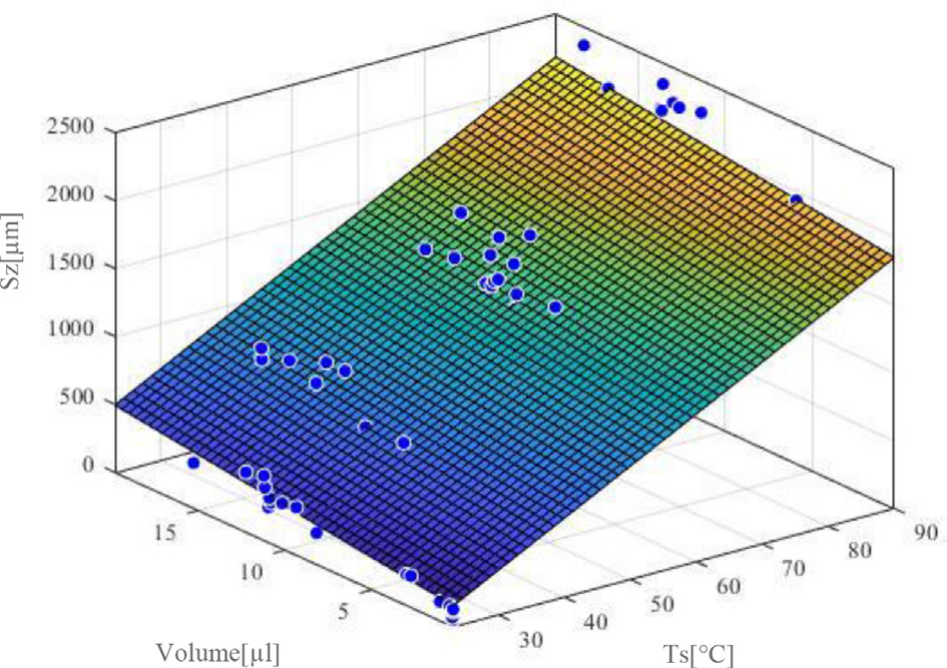
The polynomial regression plane 11 (R^2^ = 0.92) of the variation of the Sz parameter as a function of the V and Ts.

**Figure 28:**
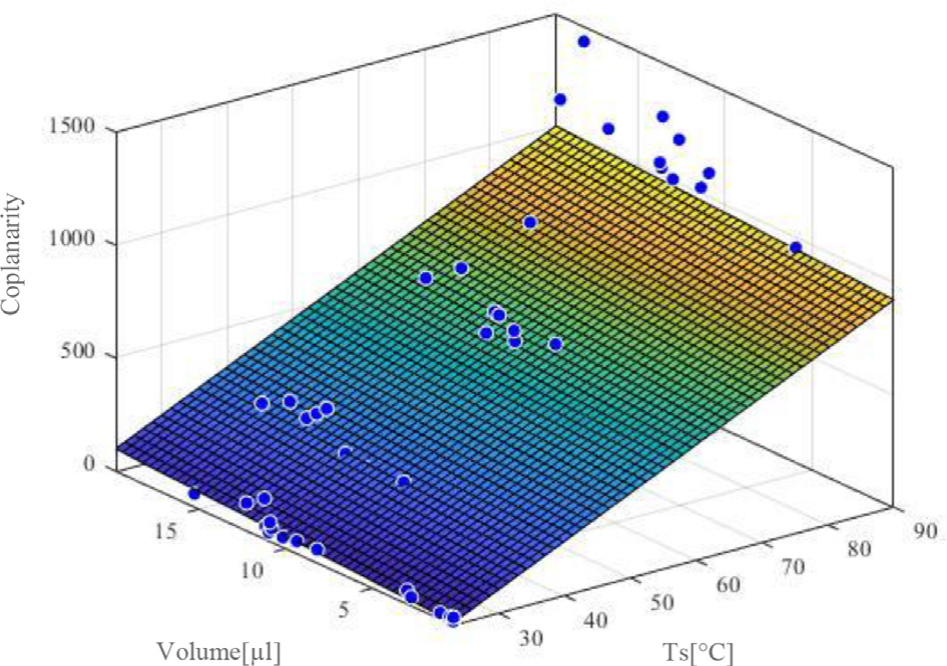
The polynomial regression plane 11 (R^2^ = 0.83) of the variation of the coplanarity parameter as a function of the V and Ts.

## References

[1] Yakhno T.A., Yakhno V.G., Sanin A.G., Sanina O.A., Pelyushenko A.S., Egorova N.A., Terentiev I.G., Smetanina S.V., Korochkina O.V., Yashukova E.V. (2005). The informative-capacity phenomenon of drying drops. IEEE Eng. Med. Biol. Mag..

[2] Sefiane K. (2010). On the formation of regular patterns from drying droplets and their potential use for bio-medical applications. J. Bionic Eng..

[3] Yakhno T.A., Sanin A.A., Ilyazov R.G., Vildanova G.V., Khamzin R.A., Astascheva N.P., Markovsky M.G., Bashirov V.D., Yakhno V.G. (2015). Drying drop technology as a possible tool for detection leukemia and tuberculosis in cattle. J. Biomed. Sci. Eng..

[4] Gorr H.M., Zueger J.M., McAdams D.R., Barnard J.A. (2013). Salt-induced pattern formation in evaporating droplets of lysozyme solutions. Colloids Surf. B Biointerfaces.

[5] Sett A., Dasgupta S., DasGupta S. (2018). Rapid estimation of the β-sheet content of Human Serum Albumin from the drying patterns of HSA-nanoparticle droplets. Colloids Surf. A Physicochem. Eng. Asp..

[6] Estes K.A., Mudawar I. (1995). Comparison of two-phase electronic cooling using free jets and sprays. J. Electron. Packag..

[7] Dan B., Wingfield T.B., Evans J.S., Mirri F., Pint C.L., Pasquali M., Smalyukh I.I. (2011). Templating of self-alignment patterns of anisotropic gold nanoparticles on ordered SWNT macrostructures. ACS Appl. Mater. Interfaces.

[8] Carreón Y.J.P., Ríos-Ramírez M., Vázquez-Vergara P., Salinas-Almaguer S., Cipriano-Urbano I., Briones-Aranda A., Díaz-Hernández O., Escalera Santos G.J., González-Gutiérrez J. (2021). Effects of substrate temperature on patterns produced by dried droplets of proteins. Colloids Surf. B Biointerfaces.

[9] Yakhno T.A., Sedova O.A., Sanin A.G., Pelyushenko A.S. (2003). On the existence of regular structures in liquid human blood serum (plasma) and phase transitions in the course of its drying. Tech. Phys..

[10] Hosseini M.-A., Nahidi F., Majdfar Z. (2007). Comparison of fern and evaporation t2ests for detection of ruptured fetal membranes Comparison of fern and evaporation tests for detection of ruptured fetal. East. Mediterr. Health J..

[11] Killeen A.A., Ossina N., McGlennen R.C., Minnerath S., Borgos J., Alexandrov V., Sarvazyan A. (2006). Protein self-organization patterns in dried serum reveal changes in B-cell disorders. Mol. Diagn. Ther..

[12] Hamadeh L., Imran S., Bencsik M., Sharpe G.R., Johnson M.A., Fairhurst D.J. (2020). Machine learning analysis for quantitative discrimination of dried blood droplets. Sci. Rep..

[13] Sobac B., Brutin D. (2011). Structural and evaporative evolutions in desiccating sessile drops of blood. Phys. Rev. E E..

[14] Brutin D., Sobac B., Loquet B., Sampol J. (2011). Pattern formation in drying drops of blood. J. Fluid Mech..

[15] Glinská G., Krajčíková K., Zakutanská K., Shylenko O., Kondrakhova D., Tomašovičová N., Komanický V., Mašlanková J., Tomečková V. (2019). Noninvasive diagnostic methods for diabetes mellitus from tear fluid. RSC Adv..

[16] Parsa M., Boubaker R., Harmand S., Sefiane K., Bigerelle M., Deltombe R. (2017). Patterns from dried water-butanol binary-based nanofluid drops. J. Nanoparticle Res..

[17] Patil N.D., Bange P.G., Bhardwaj R., Sharma A. (2016). Effects of substrate heating and wettability on evaporation dynamics and deposition patterns for a sessile water droplet containing colloidal particles. Langmuir.

[18] Kim H., Muller K., Shardt O., Afkhami S., Stone H.A. (2017). Solutal Marangoni flows of miscible liquids drive transport without surface contamination. Nat. Phys..

[19] Chen P., Harmand S., Ouenzerfi S., Schiffler J. (2017).

[20] Pyeon J., Song K.M., Jung Y.S., Kim H. (2022). Self-induced solutal Marangoni flows realize coffee-ring-less quantum dot microarrays with extensive geometric tunability and scalability. Adv. Sci..

[21] Blateyron F., Leach R. (2013). Characterisation Areal Surf. Texture.

[22] Marinello F., Pezzuolo A. (2019). Application of ISO 25178 standard for multiscale 3D parametric assessment of surface topographies. IOP Conf. Ser. Earth Environ. Sci..

[23] Kuznetsov G.V., Misyura S.Y., Volkov R.S., Morozov V.S. (2019). Marangoni flow and free convection during crystallization of a salt solution droplet. Colloids Surf. A Physicochem. Eng. Asp..

[27] Schwabe D., Rath H.J. (1992). Microgravity Fluid Mech..

[28] Snyder G. (1971). Influence of temperature and hematocrit on blood viscosity. Am. J. Physiol.-Leg. Content..

[29] Water - Density Viscosity Specific Weight | Engineering Reference and Online Tools, (n.d.). https://www.engineersedge.com/physics/water__density_viscosity_specific_weight_13146.htm (accessed October 21, 2021).

[30] Thermal Conductivity » IT’IS Foundation, (n.d.). https://itis.swiss/virtual-population/tissue-properties/database/thermal-conductivity/(accessed May 13, 2022).

[31] Tarasevich Yu.Yu., Pravoslavnova D.M. (2007). Segregation in desiccated sessile dropsof biological fluids. Eur. Phys. J. E E..

[32] Driscoll W.C. (1996). Robustness of the ANOVA and Tukey-Kramer statistical tests. Comput. Ind. Eng..

[33] Parikh V., Cheng D.-H., Linsley C., Shah K.C. (2021). Bond strength of three chairside crown reline materials to milled polymethyl methacrylate resin. J. Prosthet. Dent.

[34] Hanif A., Lu C., Chang K., Singh P., Coyner A.S., Brown J.M., Ostmo S., Chan R.V.P., Rubin D., Chiang M.F., Kalpathy-Cramer J., Campbell J.P., Chiang M.F., Ostmo S., Kim S.J., Sonmez K., Campbell J.P., Schelonka R., Coyner A., Chan R.V.P., Jonas K., Kolli B., Horowitz J., Coki O., Eccles C.-A., Sarna L., Orlin A., Berrocal A., Negron C., Denser K., Cumming K., Osentoski T., Check T., Zajechowski M., Lee T., Nagiel A., Kruger E., McGovern K., Contractor D., Havunjian M., Simmons C., Murthy R., Galvis S., Rotter J., Chen I., Li X., Taylor K., Roll K., Hartnett M.E., Owen L., Moshfeghi D., Nunez M., Wennber-Smith Z., Kalpathy-Cramer J., Erdogmus D., Ioannidis S., Martinez-Castellanos M.A., Salinas-Longoria S., Romero R., Arriola A., Olguin-Manriquez F., Meraz-Gutierrez M., Dulanto-Reinoso C.M., Montero-Mendoza C. (2022).

